# Anxiety and the development and maintenance of anorexia nervosa: protocol for a systematic review

**DOI:** 10.1186/s13643-018-0685-x

**Published:** 2018-01-24

**Authors:** E. Caitlin Lloyd, Anne M. Haase, Bas Verplanken

**Affiliations:** 1 Centre for Exercise, Nutrition and Health Sciences, University of Bristol, Bristol, UK; 2 Department of Psychology, University of Bath, Bath, UK

**Keywords:** Aetiology, Anxiety, Anorexia nervosa: risk factor, Longitudinal studies

## Abstract

**Background:**

Several aetiological models of anorexia nervosa (AN) hold non-eating/weight-gain-related anxiety as a factor relevant to the onset and maintenance of the disorder. Longitudinal studies that allow assessment of this hypothesis have been conducted; however, the evidence has not yet been aggregated in a systematic manner. The proposed study will systematically review articles describing prospective investigations of the relationship between anxiety and AN development or maintenance, with the aim of providing a balanced summary of current understanding and identifying areas for further research.

**Methods/design:**

Electronic databases will be searched for articles investigating the longitudinal influence of non-eating/weight-gain-related anxiety (anxiety disorders and trait anxiety) on the development/maintenance of AN. References of eligible articles will be searched to ensure the identification of all relevant studies. Two independent reviewers will complete the title and abstract, and full-text, screening, with a third independent reviewer resolving any conflicts at each stage. A systematic review will be completed, and the quality of the included studies, as well as the strength of the body of evidence generated, will be assessed and reported.

**Discussion:**

Although there are limitations to the present review, understanding the current evidence for the role of non-eating/weight-gain-related anxiety in AN can direct future research that may ensure accurate aetiological models of AN and effective treatments.

**Systematic review registration:**

The study is registered on PROSPERO under the reference number CRD42017069644

**Electronic supplementary material:**

The online version of this article (10.1186/s13643-018-0685-x) contains supplementary material, which is available to authorized users.

## Background

Anorexia nervosa (AN) is characterised by the maintenance of a significantly low body weight [1] that is achieved by dietary restriction that persists despite severe risks to physical health [2]. The factors promoting the onset and continuation of excessive and pathological starvation are poorly understood, preventing such factors being targeted by treatment interventions [3, 4]. As a result, the recovery rate of AN is low, the relapse rate is high, and the mortality rate is the greatest of any psychiatric illness [5, 6].

The high levels of anxiety surrounding eating has caused AN to be compared to anxiety disorders [3], and anxious symptomatology in AN has been empirically investigated in attempts to better understand AN aetiology. Supporting clinical observations of elevated anxiety in individuals with AN, it is consistently reported that individuals who experience AN are more likely to have anxiety disorders and greater levels of anxious symptomatology and trait anxiety, compared to the general population [7–9]. This holds prior to the illness, at the time of AN and in recovery [7]. Subsequently, a number of models of illness have proposed anxiety as a key factor in the development and maintenance of AN. It is proposed that the high levels of anxiety individuals with AN experience cause starvation to be particularly valuable, given dietary restriction affects neurobiological systems that appear to modulate anxiety in AN [10–12].

The relief of anxiety caused by dietary restriction is suggested to encourage excessive engagement in the behaviour [10–12]. It is asserted that individuals with AN come to depend on dietary restriction to avoid extreme levels of anxiety, with this anxiety increasingly focused on food and weight gain. Thus, when anxiety is greater, it would be expected that restrictive eating is more likely to occur in AN. Pre-meal anxiety is related to reduced caloric intake at the meal [13], and individuals with AN are more likely to report restricting their intake at times that coincide with high levels of state anxiety [14, 15]. This suggests that traits and disorders that cause episodes characterised by high state anxiety, such as anxiety disorders and trait anxiety, would be negative prognostic factors for AN, state anxiety serving to increase engagement in dietary restriction.

An alternative hypothesis is that anxious traits and anxiety disorders reflect underlying neurobiological abnormalities that predispose individuals to develop pathological fear and avoidant responses [7, 16, 17]. The abnormalities result in the development of fears and abnormal behaviour surrounding weight gain and eating when weight concern is present and dieting is initiated. In these models, it is only anxiety surrounding eating and weight gain that is directly associated with continued dietary restriction. Non-eating/weight-gain concerns are factors predictive of the onset and maintenance of AN because they reflect the severity of eating/weight-related anxiety and of eating behaviours designed to manage this anxiety.

The relationship of trait anxiety and symptoms/diagnoses of anxiety disorders (i.e. stable forms of anxiety that do not specifically relate to eating/weight gain) with both AN onset and maintenance has been probed in a number of longitudinal studies. However, the evidence gathered across these studies has not yet been synthesised in a systematic manner. This prevents a fair evaluation of the longitudinal relationship between anxiety and AN, which is necessary for theoretical accounts of AN, and disorder prevention/treatment interventions, to be appropriately informed. It also means that the need for further investigation in this area may not be fully appreciated. This systematic review will gather and organize evidence from a diverse range of observational studies, to critically evaluate the nature of the relationship between non-eating/weight gain-related anxiety, which we refer to as anxiety in this manuscript, and AN. The current protocol outlines the methods of our investigation, in accordance with the PRISMA-P checklist (available in Additional file 1), which will address the following research questions:

1. Is anxiety related to the later onset of AN?
2. Is anxiety related to the maintenance of AN?

## Methods

### Eligibility criteria

#### Study designs

Retrospective and prospective cohort and case-control studies that present original data and that investigated the longitudinal relationship between anxiety and AN development or maintenance will be eligible for inclusion in the review. Studies must have measured anxiety at one time point and have assessed AN symptoms at least 1 year later to be included.

#### Participants

We will only include studies that include a human AN population, and individuals in the AN sample recruited must meet, or have previously met, full diagnostic criteria for the disorder.

#### Exposures

Eligible exposures are symptoms/diagnosis of any anxiety disorder (excluding obsessive-compulsive disorder or posttraumatic stress disorder) and trait anxiety, provided they have been assessed with a validated measure. Studies that have assessed relationships between state anxiety and AN pathology will not be included given we are considering the relevance of more stable forms of anxiety to AN. Further, we are specifically interested in the relationship between non-eating/weight gain-related anxiety and AN, and state anxiety assessments are likely to capture anxiety related to eating and weight gain in individuals with AN. While the range of eligible exposures may seem broad, an initial scoping review of the literature suggests this approach is reasonable and will not result in an unmanageable amount of data to synthesise.

#### Comparators

Studies may compare individuals with anxiety disorders, or high levels of anxiety disorder symptoms/trait anxiety, with individuals without these disorders/characteristics.

#### Outcomes

For studies investigating the relationship between anxiety and the development of AN, AN diagnosis is the primary outcome. Secondary outcomes are severity of behavioural/psychological symptoms of AN and body mass index (BMI).

For studies investigating the relationship between anxiety and AN maintenance, the primary outcome is recovery from AN. Secondary outcomes are severity of behavioural/psychological symptoms of AN/changes in these symptoms and BMI/change in BMI.

#### Timing

Outcome data may be collected at different time points; however, AN onset or maintenance must be assessed at least 1 year after the time at which anxiety is measured for the study to be included.

#### Setting

We impose no restrictions pertaining to study setting.

#### Language

Only studies reported in English will be included.

#### Information sources

Literature searches will be conducted on articles held in Medline and PsychInfo using the OVID interface. To identify relevant articles in these databases, a search strategy has been produced and is available in Additional file 2. Eligible studies will have been published in a peer-reviewed journal and in the year 1980 or subsequently. Reference lists of eligible studies identified from database searches will be scanned to ensure we capture all relevant research articles.

### Data collection and analysis

#### Study selection

Following removal of duplicates, the titles and abstracts of studies retrieved using the database searches will be screened by two reviewers. The full text of potentially eligible studies will be retrieved and assessed for inclusion in the review by two reviewers. Should the full text of a study not be accessible through institutional memberships study authors will be contacted in order to retrieve the manuscript. The decision to include studies will be based on criteria outlined in the preceding section, and a third reviewer will resolve any discrepancies between the screeners at both stages. Two reviewers must also approve the inclusion of further studies identified from reference lists of the eligible articles found using database searches. The reason for exclusion of any study will be recorded, and the study selection process presented in a PRISMA flow diagram.

#### Data extraction

Using a tailored data collection form, the following information will be extracted from each study:

1. Publication details: authors, title, publication date, and country.
2. Study information: study setting and design, details of exposure and outcome, details of comparator (if appropriate), sample size at recruitment and completion, follow-up period, and study measures.
3. Participant characteristics: demographics, illness duration, BMI, number of hospitalisations, length of illness, psychiatric co-morbidities, and use of psychotropic medication.
4. Study results: findings in relation to the primary and secondary outcomes will be reported, as will the statistical methods employed in the investigation.

Where data are missing, we will attempt to contact study authors to obtain this.

#### Risk of bias

The National Institute of Health’s Quality Assessment Tool for Observational Cohort and Cross-Sectional Studies [18] will be used to assess risk of bias for each study. The checklist considers selection bias, blinding of outcome assessors (researcher bias), withdrawal (attrition bias), and selective reporting (reporting bias). Other aspects of study quality are also considered: the validity of exposure and outcome measures, risk of confounding, sample size, and potential to capture dose-response relationships. Regarding the validity of exposure measures, we are particularly concerned with evaluating the potential for these to capture anxiety that reflects the AN (i.e. that related to eating and weight gain). For example, social phobia assessments may capture fears of eating in public, which could be symptomatic of the AN rather than of social phobia. This would give rise to false inferences concerning the relationship between non-eating/weight gain-related anxiety and AN.

Risk of bias and quality for all studies will be assessed by two reviewers, with discrepancies resolved by a third reviewer, to ensure reliability of the review. If eligible review studies are identified, the outcomes of these review studies will be compared to the outcomes reported by articles included in the present review, to detect systematic reporting biases. The strength of the body of evidence collected in the course of the review will be assessed using the Grading of Recommendations Assessment, Development and Evaluation (GRADE) system [19].

### Data synthesis

Studies will be categorised according to whether they assess the relationship of anxiety with the development or the maintenance of AN. Within these two categories, studies will be grouped further, according to the type of anxiety considered for its influence on AN (i.e. specific anxiety disorders and trait anxiety). Ideally, meta-analyses would be conducted to determine the pooled effect size pertaining to the longitudinal relationship of each type of anxiety with both AN onset and AN maintenance. However, preliminary investigations suggest that the number of eligible studies within each category, and the heterogeneity of these studies, will mean meta-analyses are not feasible.

A systematic narrative review will describe findings of the included studies, and the similarities and differences between studies, for studies grouped by outcome (AN onset or maintenance) and type of anxiety measured. A table outlining findings of each study will also be provided. RevMan version 5.3 [20] will be used to handle and synthesise the data of the included studies, for completion of the qualitative review.

## Discussion

With the inclusion of anxiety in aetiological models of AN, it is important that the relevance of anxiety to AN development and maintenance is clarified. This study will provide the first, much needed, systematic synthesis of longitudinal studies that investigate the relationship between anxiety and AN. By considering a range of anxiety exposures (i.e. anxiety disorder diagnoses/symptoms and trait anxiety), we hope to be able to evaluate a sufficient number of studies for our research questions to be addressed.

The planned quality assessment is extensive, to enable a comprehensive evaluation of the risk of bias within and across included studies, promoting the validity of conclusions arising from the review. The extent of the data extracted from studies will allow for the identification of differences in findings that may arise from participant and study characteristics. Most importantly, our method is transparent and explicitly outlined in detail, allowing its replication, as well as assessment of its quality, by others.

Anticipated challenges lie in collecting a sufficient number of studies for firm conclusions regarding the relationship between particular types of anxiety and AN onset/maintenance to be made. Interpreting the body of evidence surrounding AN maintenance may also be problematic given the definitions of recovery are not standardised in the field of eating disorders [21]. We consider the outcome of AN recovery, as opposed to remission or relapse, in an attempt to focus the research question. However, understanding the relationship of anxiety with both remission and relapse in AN would further inform knowledge of the factors associated with AN maintenance.

Because of their longitudinal nature, studies of the review are likely to be subject to high levels of attrition, which is an issue that has implications on the validity of conclusions that may be drawn from the review. A further limitation is that we will be unable to parse apart the explanatory power of different types of anxiety on AN development/maintenance given studies may have measured only one type of anxiety, focusing on one disorder or trait anxiety for example. Alternatively, studies may not have considered the unique contribution of each type of measured anxiety to AN risk. The review will not be able to determine whether non-eating/weight gain-related anxiety directly causes dietary restriction, or if such reflects an underlying process that promotes concern surrounding eating and weight gain. However, findings can inform the value of continuing to study anxiety that does not surround eating and weight gain in relation to AN.

Despite the discussed limitations, it is important for evidence surrounding the longitudinal influence of anxiety on AN to be synthesised so that progress with investigation and understanding may be presented. By highlighting areas that require further study, the review may encourage the development of accurate aetiological models of AN, which may inform effective treatment.

## Additional files

Additional file 1: PRISMA-P 2015 Checklist. Checklist of recommended items to address in a systematic review protocol. (DOCX 38 kb)

Additional file 2: Systematic Review Search Strategy. Details of the search strategy used to identify relevant articles in the databases Medline and PsychInfo. (DOC 28 kb)

## Abbreviations

AN: Anorexia nervosa
BMI: Body mass index
GRADE: Grading of Recommendations Assessment, Development and Evaluation
PRISMA: Preferred Reporting Items for Systematic Reviews and Meta-Analyses

## References

[1] American Psychiatric Association. Diagnostic and statistical manual of mental disorders: DSM-5. Washington, DC: American Psychiatric Association; 2013.

[2] Walsh BT (2013). The enigmatic persistence of anorexia nervosa. Am J Psychiatry.

[3] Guarda AS, Schreyer CC, Boersma GJ, Tamashiro KL, Moran TH (2015). Anorexia nervosa as a motivated behavior: relevance of anxiety, stress, fear and learning. Physiol Behav.

[4] Kleiman SC, Watson HJ, Bulik-Sullivan EC, Huh EY, Tarantino LM, Bulik CM, Carroll IM (2015). The intestinal microbiota in acute anorexia nervosa and during renourishment: relationship to depression, anxiety, and eating disorder psychopathology. Psychosom Med.

[5] Steinhausen HC. The outcome of anorexia nervosa in the 20th century. Am J Psychiatry. 2002;159(8):1284-93. 10.1176/appi.ajp.159.8.1284 12153817

[6] Arcelus J, Mitchell AJ, Wales J, Nielsen S (2011). Mortality rates in patients with anorexia nervosa and other eating disorders. A meta-analysis of 36 studies. Arch Gen Psychiatry.

[7] Steinglass JE, Sysko R, Glasofer D, Albano AM, Simpson HB, Walsh BT (2011). Rationale for the application of exposure and response prevention to the treatment of anorexia nervosa. Int J Eat Disord.

[8] Wagner A, Barbarich-Marsteller NC, Frank GK, Bailer UF, Wonderlich SA, Crosby RD (2006). Personality traits after recovery from eating disorders: do subtypes differ?. Int J Eat Disord.

[9] Kaye WH, Bulik CM, Thornton L, Barbarich N, Masters K, Price Fdn Collaborative G (2004). Comorbidity of anxiety disorders with anorexia and bulimia nervosa. Am J Psychiatr.

[10] Kaye W (2008). Neurobiology of anorexia and bulimia nervosa. Physiol Behav.

[11] Nunn K, Frampton I, Lask B (2012). Anorexia nervosa—a noradrenergic dysregulation hypothesis. Med Hypotheses.

[12] Lloyd EC, Frampton I, Verplanken B, Haase AM (2017). How extreme dieting becomes compulsive: a novel hypothesis for the role of anxiety in the development and maintenance of anorexia nervosa. Med Hypotheses.

[13] Steinglass JE, Sysko R, Mayer L, Berner LA, Schebendach J, Wang YJ (2010). Pre-meal anxiety and food intake in anorexia nervosa. Appetite.

[14] Haynos AF, Crosby RD, Engel SG, Lavender JM, Wonderlich SA, Mitchell JE (2015). Initial test of an emotional avoidance model of restriction in anorexia nervosa using ecological momentary assessment. J Psychiatr Res.

[15] Lavender JM, De Young KP, Wonderlich SA, Crosby RD, Engel SG, Mitchell JE (2013). Daily patterns of anxiety in anorexia nervosa: associations with eating disorder behaviors in the natural environment. J Abnorm Psychol.

[16] Strober M (2004). Pathologic fear conditioning and anorexia nervosa: on the search for novel paradigms. Int J Eat Disord.

[17] Strober M, Freeman R, Lampert C, Diamond J (2007). The association of anxiety disorders and obsessive compulsive personality disorder with anorexia nervosa: evidence from a family study with discussion of nosological and neurodevelopmental implications. Int J Eat Disord.

[18] National Heart Lung and Blood Institute. Quality Assessment Tool for Observational Cohort and Cross-Sectional Studies - NHLBI, NIH. [online] Nhlbi.nih.gov. 2014. Available at: https://www.nhlbi.nih.gov/health-topics/study-quality-assessment-tools. Accessed 12 Nov 2017.

[19] The GRADE working group. GRADE home. 2017. [online] Available at: http://www.gradeworkinggroup.org/. Accessed 12 Nov 2017.

[20] Review Manager (RevMan) [Computer program]. Version 5.3. Copenhagen: The Nordic Cochrane Centre, The Cochrane Collaboration; 2014.

[21] Khalsa SS, Portnoff LC, McCurdy-McKinnon D, Feusner JD (2017). What happens after treatment? A systematic review of relapse, remission, and recovery in anorexia nervosa. J Eat Disord.

