# Supplementary material for: Anxiety and the development and maintenance of anorexia nervosa: protocol for a systematic review
Source: Syst Rev. 2018 Jan 24;7:14. doi: 10.1186/s13643-018-0685-x (PMC5782391; doi:10.1186/s13643-018-0685-x)
Supplement: Supplementary file 2 — Systematic Review Search Strategy. Details of the search strategy used to identify relevant articles in the databases Medline and PsychInfo. (DOC 28 kb) [file 13643_2018_685_MOESM2_ESM.doc]

Additional file 2

**Table S2. Search Terms**

1. anorexi*.tw.
2. exp Anorexia Nervosa/ or exp Anorexia/
3. Anxiety Disorders/ or Anxiety/
4. anxiety.tw.
5. antecedent
6. risk factor*.tw.
7. Risk Factors/
8. recover*.tw.
9. prospective*.tw.
10. retrospective*.tw.
11. case control.tw
12. cohort stud*.tw
13. Prospective Studies/mt [Methods]
14. Retrospective Studies/mt [Methods]
15. Longitudinal Studies/mt [Methods]
16. longitudinal*.tw.
17. follow-up.tw.
18. 9 or 10 or 11 or 12 or 13 or 14 or 15 or 16 or 17
19. 5 or 6 or 7 or 8
20. 3 or 4
21. 1 or 2
22. cancer*.tw.
23. 18 and 19 and 21
24. 19 and 20 and 21
25. 23 or 24
26. 25 not 22
27. limit 26 to english language
28. 27 and "Journal Article".sa_pubt.
29. limit 28 to yr="1980 - 2017"
